# High cystatin C levels predict long‐term mortality in patients with ST‐segment elevation myocardial infarction undergoing late percutaneous coronary intervention: A retrospective study

**DOI:** 10.1002/clc.23179

**Published:** 2019-04-09

**Authors:** Yuewu Chen, Yan Fan, Min Men, Guidong Shen, Aiqun Ma

**Affiliations:** ^1^ Department of Cardiovascular Medicine First Affiliated Hospital of Xi'an Jiaotong University Xi'an China; ^2^ Department of Cardiovascular Medicine The Second Affiliated Hospital of Hainan Medical University Haikou China; ^3^ Department of Cardiovascular Medicine Gansu Provincial Hospital Lanzhou China; ^4^ Department of Endocrinology Xi'an Central Hospital Xi'an China; ^5^ Department of Cardiovascular Medicine Ankang Central Hospital AnKang China

**Keywords:** cystatin C, late percutaneous coronary intervention, prognosis, ST‐segment elevation myocardial infarction

## Abstract

**Objectives:**

Late percutaneous coronary intervention (PCI) in patients with ST‐segment elevation myocardial infarction (STEMI), defined as time of PCI > 7 days from symptom onset, is a common practice with clinical benefits. This study aimed to evaluate the predictive value of admission cystatin C (cys C) level on long‐term mortality in STEMI patients receiving late PCI.

**Methods:**

Medical records of STEMI patients who were hospitalized between 2009 and 2011 from eight PCI‐capable hospitals in Northwest China were retrospectively analyzed. Cys C level ≥ 1.105 mg/L was considered as the best predictor of long‐term mortality based on the receiver‐operating characteristic analysis. Patients were followed up by phone or face‐to‐face interviews, and the long‐term mortality was obtained by reviewing medical records.

**Results:**

The final analysis included 716 STEMI patients who received late PCI and had available cys C levels prior to PCI, and 524 were assigned into the high cys C group and 192 the low cys C group. Patients were followed up for an average length of 40.37 months. Compared with the low cys C group, the high cys C group had a higher long‐term all‐cause mortality (10.4% vs 2.9%, *P* < 0.001) and a higher cardiac mortality (6.8% vs 2.1%, *P* = 0.004). Multivariate Cox regression analysis showed that high cys C level was an independent predictor for both long‐term all‐cause mortality and cardiac mortality.

**Conclusions:**

High cys C level at admission is an independent predictor of long‐term mortality in STEMI patients undergoing late PCI.

## 1 INTRODUCTION

ST‐segment elevation myocardial infarction (STEMI) is one of the three types of acute myocardial infarction, and remains a leading cause of morbidity and mortality worldwide, with approximately 12% patients died within 6 months.^1^ In‐hospital mortality in patients of unselected STEMI decreased with the use of timely and effective percutaneous coronary intervention (PCI), reperfusion therapy, and modern antithrombotic therapy.^1^, ^2^ Early mechanical (PCI) or pharmacological reperfusion has been the best method for treating patients with clinical manifestations of STEMI within 12 hours of symptom onset. However, the large proportion of late presenters fall out of the initial 12‐hour optimal treatment time window. Clinically, late PCI, defined as the time to open an infarct‐related artery (IRA) from symptoms onset >7 days (when the myocardial condition is considered stable), is practiced commonly for these late presenters. Whether late PCI is adequately beneficial is controversial. In our previous study, late PCI in STEMI patients prevented cardiac remodeling and improved clinical outcomes.^3^, ^4^ Therefore, the exploration of effective prognostic factors for the late PCI patients is of clinical significance, and in this context, cystatin C (cys C) is proposed to be a potential prognostic factor in patients with STEMI who have undergone primary PCI in the literature.^5^

Cys C, produced by all cells with a nucleus, is excreted into blood and freely filtered by the glomerular.^6^, ^7^ Cys C is also released from myocardial cells and hypoxia increases its production.^8^ As a potent inhibitor of lysosomal proteinases and cysteine proteases, cys C modulates inflammatory response, phagocytic functions and extracellular matrix degradation. Cys C is considered to be an ideal biomarker for glomerular filtration rate (GFR).^9^ Recently, cys C has been found to be able to predict new‐onset or deteriorating cardiovascular disease,^10^ and in patients with STEMI undergoing primary PCI, cys C has shown prognostic significance.^5^ However, in patients with STEMI undergoing late PCI, the relationship between admission cys C levels and long‐term outcomes remains unknown. This study aimed to investigate the prognostic value of admission cys C in predicting long‐term mortality in STEMI patients undergoing late PCI.

## 2 METHODS

### 2.1 Study protocol

This study is a retrospective and multicenter observational study. We retrospectively reviewed the medical records of STEMI patients underwent late PCI in 8 PCI‐capable hospitals in Northwest China from January 2009 to December 2011. STEMI was diagnosed according to the 2007 American College of Cardiology Foundation/American Heart Association guidelines^11^: Persistent ischemic symptoms for ≥30 minutes; ST‐segment elevation ≥1 mm in at least 2 adjacent limb leads or ≥ 2 mm in at least 2 contiguous precordial leads, or the presence of a new or suspicious new left bundle branch block, or the development of pathological Q waves; creatine kinase and creatine kinase‐myocardial band elevated ≥ two times the upper limit of normal, or elevated cardiac troponins. The study protocol was approved by the Ethics Committee of the First Affiliated Hospital of Xi'an Jiaotong University School of Medicine. The ethics committee waived the need to obtain informed consents because it would be impracticable to obtain written consent from patients, because that the study investigated mortality and many of the patients who admitted hospitals during 2009 to 2011 died by the time of the study conduction.

### 2.2 Inclusion and exclusion

The medical records of patients with STEMI underwent PCI were accessed and retrieved from eight PCI‐capable hospitals in Northwest China. The hospitalization dates extended from January 2009 to December 2011. The medical records include baseline demographic data, laboratory findings, clinical diagnosis, treatment strategies, and clinical outcomes. All included patients were diagnosed with STEMI and had undergone a late PCI (defined as the time to open IRA from symptom onset >7 days). Patients were excluded if they underwent an early PCI, or had diagnosed with idiopathic cardiomyopathy, congenital heart disease, valvular heart disease, rheumatic or autoimmune disease, tumors, severe liver or kidney dysfunction, or other systemic diseases.

### 2.3 Variables and clinical endpoints

We intended to find effective prognostic factors for late PCI patients through reviewing their medical records, and the screened potential predictors including routine blood test such as blood lipids, blood glucose, liver and kidney function, and b‐type natriuretic peptide (BNP). Cys C was selected as the best candidate for prognostic factors based on primary analysis and literature review. Patients were further grouped into the high cys C group and the low cys C group according to the receiver‐operating characteristic analysis. The primary endpoint was designated to be the long‐term all‐cause mortality and long‐term cardiac mortality. This information was obtained by reviewing hospital records. Cardiac death includes deaths caused by acute myocardial infarction, heart failure, heart attack, or arrhythmia.

### 2.4 Laboratory analysis

Before PCI, blood was sampled within 1 hour after admission. Serum cys C was measured by colloidal gold particle‐enhanced colorimetric immunoassay (Nescauto GC Cystatin C, Alfresa Pharma, Osaka, Japan) with a Hitachi 7600‐110 automatic analyzer. Simplified Modification of Diet in Renal Disease (MDRD) equation was used to calculate the estimated glomerular filtration rate (eGFR). Based on the receiver‐operating characteristic analysis, the cys C level ≥ 1.105 mg/L was considered to be the best predictor of long‐term mortality. According to the cutoff value, patients were divided into the high and the low cys C groups.

### 2.5 Statistical analysis

**Figure 1:**
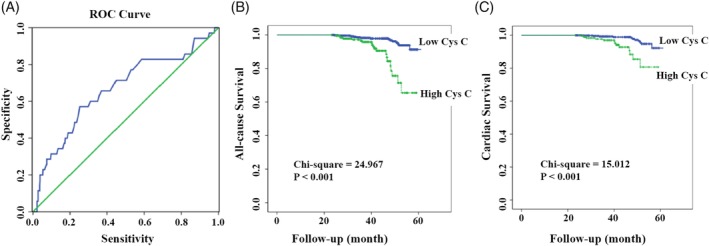
A**,** Receiver‐operator characteristic (ROC) curve of the optimal cutoff value of admission cys C for predicting long‐term mortality. B, Kaplan‐Meier survival curves for long‐term all‐cause mortality. C**,** Kaplan‐Meier survival curves for long‐term cardiac mortality

Continuous variables are expressed as mean ± SD or median and interquartile range (M [Q1‐Q3]). Categorical variables are expressed as frequencies and percentages. Independent‐ sample Student's *t* test or Mann‐Whitney *U* test was used to compare the differences between continuous variables. The Pearson χ^2^ test or the Fisher exact test were used to compare the differences between categorical variables. Receiver‐operator characteristic curve analysis was used to determine the cutoff value of cys C for predicting long‐term mortality (Figure 1A). The Kaplan‐Meier method was used to construct the cumulative survival curves for long‐term all‐cause mortality and long‐term cardiac mortality. Multivariate Cox regression was used to identify the independent risk factors for long‐term mortality during follow‐up. All statistical tests were two‐tailed and a *P*‐value <0.05 was considered statistically significant. All statistical analyses were performed using SPSS v.18.0 (SPSS Inc., Chicago, Illinois).

## 3 RESULTS

### 3.1 Baseline patient characteristics

A total of 716 STEMI patients were included for the final analysis. The screening of patients was as follows: First, medical records of 1887 patients with STEMI and underwent PCI were obtained and retrieved from eight PCI‐capable hospitals in Northwest China. Second, patient who received intervention other than a late PCI were excluded. Third, patients without available cys C level at admission were excluded. According to the ROC curve analysis, 1.105 mg/L was chosen as the best cutoff for prediction of long‐term mortality (area under the curve = 0.67, sensitivity *=* 57%, specificity *=* 76%) (Figure 1). On the basis of the criterion, the patients were divided into low cys C group (<1.105 mg/L, n = 524) and high cys C group (>1.105 mg/L, n = 192).

The baseline characteristics of the patients are summarized in Table 1. Patients in high cys C group were older (*P* < 0.001), had a higher heart rate at admission (*P* = 0.049) and a higher incidence of hypertension (*P* = 0.003). The Killip classification at admission was significantly different between the two groups: the frequency of patients with Killip classification Class I was lower and with Class II, III, or IV was higher in the high cys C group. Gender at admission showed no difference between the two groups. The left ventricular ejection fraction (LVEF) showed no difference between the two groups, however, for LVEF <45%, there was a difference between the two groups (*P* = .350.008) (Table 1).

**Table 1:** Baseline characteristics and angiographic characteristics of patients

Variable	Low cys C group (<1.105 mg/L)	High cys C group (≥1.105 mg/L)	*P‐*value
Age (years)	55.34 ± 10.41	63.49 ± 10.50	<0.01
Male sex	447 (85.3%)	160 (82.5%)	0.206
Body mass index (kg/m^2^)	24.06 ± 2.89	23.51 ± 2.87	0.024
HR on admission (bpm)	74.3 ± 13.59	76.63 ± 15.44	0.049
SBP on admission (mm Hg)	120.66 ± 18.82	123.12 ± 20.58	0.131
DBP on admission (mm Hg)	76.59 ± 12.27	76.81 ± 12.76	0.835
Smoking	357 (68.1%)	134 (69.1%)	0.442
Alcohol consumption	167 (31.9%)	66 (34.0%)	0.323
Hypertension	205 (39.1%)	99 (51.0%)	0.003
Diabetes mellitus	74 (14.1%)	24 (12.4%)	0.318
Hyperlipidemia	92 (17.6%)	30 (15.5%)	0.294
Prior revascularization	15 (2.9%)	6 (3.1%)	0.520
LVEF classification			0.070
LVEF ≥55%	251 (47.9%)	81 (42.2%)	
LVEF (45%‐54%)	169 (32.3%)	55 (28.6%)	
LVEF <45%			0.008
LVEF (30%‐44%)	99 (18.9%)	53 (27.6%)	
LVEF <30%	5 (1.0%)	3 (1.6%)	
Killip classification on admission			<0.001
I	337 (64.3%)	96 (49.5%)	
II	152 (29.0%)	70 (36.1%)	
III	30 (5.7%)	18 (9.3%)	
IV	5 (1.0%)	10 (5.2%)	
Myocardial infarction location			
Anterior/anteroseptal	295 (56.3%)	117 (60.3%)	0.189
Lateral	17 (3.2%)	4 (2.1%)	0.288
Inferior/posterior/right ventricular	248 (47.3%)	85 (43.8%)	0.226
Culprit lesion (coronary)			0.460
Right	191 (36.5%)	59 (30.4%)	
Left anterior descending	283 (54.0%)	114 (58.8%)	
Left circumflex	45 (8.6%)	18 (9.3%)	
Left main	5 (1.0%)	3 (1.5%)	
TIMI flow grade pre‐PCI			0.908
0	159 (30.3%)	54 (27.8%)	
1	16 (3.1%)	7 (3.6%)	
2	40 (7.6%)	16 (8.2%)	
3	309 (59.0%)	117 (60.3%)	
Number of narrowed coronary vessels			0.562
1	151 (28.8%)	49 (25.3%)	
2	179 (34.2%)	66 (34.0%)	
≥3	194 (37.0%)	79 (40.7%)	

Abbreviations: DBP, diastolic blood pressure; HR, heart rate; LVEF, left ventricular ejection fraction; PCI, percutaneous coronary intervention; SBP, systolic blood pressure; TIMI, thrombolysis in myocardial infarction.

Data are presented as the mean ± SD, median (IQR), or n (%).

### 3.2 Laboratory findings

Baseline laboratory data for patients are listed in Table 2. N‐terminal (NT)‐pro hormone BNP (NT‐proBNP) in the high cys‐C group was higher than in the low cys C group (1366.00 (788.20‐3019.00) vs 943.20 (423.05‐1711.00), *P* < 0.001), same as serum creatinine (95.68 ± 21.51 vs 82.15 ± 13.44, *P* < 0.001). The eGFR of the high cys C group was 73.12 ± 16.58 and the low cys C group was 88.91 ± 16.42 (*P* < 0.001), and the kidney function of patients in both group were mildly reduced. Patients with high Cys C had a lower creatine kinase‐MB than patients with a low cys C (17.40 (10.90‐49.55) vs 22.55 (11.29‐79.64), *P* = 0.037) (Table 2).

**Table 2:** Laboratory findings of patients at admission

Variable	Low cys C (<1.105 mg/L) group	High cys C (≥1.105 mg/L) group	*P‐*value
White blood cells (10^9^/L)	8.69 ± 3.69	8.06 ± 3.23	0.038
Neutrophil (%)	68.26 ± 15.11	67.79 ± 14.30	0.708
Hemoglobin (g/L)	135.80 ± 15.95	132.02 ± 17.07	0.006
Platelet (10^9^/L)	195.39 ± 63.90	193.45 ± 83.62	0.740
Total cholesterol (mmol/L)	4.08 ± 1.07	3.83 ± 1.05	0.005
Triglycerides (mmol/L)	1.77 ± 1.00	1.50 ± 0.80	0.001
High‐density lipoprotein (mmol/L)	1.00 ± 0.24	1.01 ± 0.22	0.440
Low‐density lipoprotein (mmol/L)	2.41 ± 0.87	2.15 ± 0.70	<0.001
Estimated glomerular filtration rate (eGFR)^a^ (mL/min/1.73 m^2^)	88.91 ± 16.42	73.12 ± 16.58	<0.001
Serum creatinine (μmol/L)	82.15 ± 13.44	95.68 ± 21.51	<0.001
Glucose (mmol/L)	7.37 ± 3.06	6.49 ± 2.88	0.001
Uric acid (μmol/L)	287.46 ± 78.85	322.94 ± 95.54	<0.001
Creatine kinase‐MB (U/L)	22.55 (11.29–79.64)	17.40 (10.90‐49.55)	0.037
Tropinin I (ng/mL)	1.11 (0.55‐3.31)	1.00 (0.54‐3.54)	0.837
NT‐proBNP (pg/mL)	943.20 (423.05–1711.00)	1366.00 (788.20–3019.00)	<0.001
Cys C (mg/L)	0.85 ± 0.15	1.75 ± 0.17	<0.001

Estimated glomerular filtration rate (eGFR) was calculated by the abbreviated Modification of Diet in Renal Disease equation: eGFR (mL/min/1.73 m^2^ of body surface area) = 186 × (Serum creatinine/88.4)^−1.154^ × (Age)^−0.203^ (×0.742 for females). Data are presented as the mean ± SD, median (IQR), or n (%).

a eGFR reference range: ≥90, Normal range, 60‐89: mildly reduced renal function.

### 3.3 Follow‐up outcomes

The mean follow‐up period was 40.37 months. The long‐term all‐cause mortality (10.4% vs 2.9%, *P* < 0.001) and the long‐term cardiac mortality (6.8% vs 2.1%, *P* = 0.004) were significantly higher in the high cys C group, as compared to the low cys C group (Table 3). The survival curves for all‐cause mortality and cardiac mortality are shown in Figure 1B and C, respectively. The survival curves showed that the long‐term cumulative survival for all‐cause mortality (χ^2^ = 24.967, *P* < 0.001) and cardiac mortality (χ^2^ = 15.012, *P* < 0.001) were significantly lower in the high cys C group.

**Table 3:** Long‐term cardiac events

Variable	Low cys C group (<1.105 mg/L)	High cys C group (≥.105 mg/L)	*P*‐value
All‐cause mortality	15 (2.9%)	20 (10.4%)	<0.001
Cardiac mortality	11 (2.1%)	13 (6.8%)	0.004

Data are presented as n (%).

### 3.4 Multivariate regression analysis

According to the multivariate analysis, high cys C level, age, and admission heart rate were independent predictor of both long‐term all‐cause mortality (cys C, odds ratio [OR] 1.51, 95% confidence interval [CI] 1.30‐1.75, *P* < 0.001; age, OR 1.07, 95% CI 1.04‐1.11, *P* < 0.001; hazard ratio [HR] at admission, OR 1.02, 95% CI 1.00‐1.04, *P* = 0.043) and cardiac mortality (cys C, OR 1.36, 95% CI 1.20‐1.53, *P* < 0.001; age, OR 1.05, 95% CI 1.00‐1.09, *P* = 0.019; HR at admission, OR 1.03, 95% CI 1.00‐1.05, *P* = 0.036) (Table 4, non‐significant variables are not listed). The creatinine level at admission and the eGFR were significantly different between the two groups. None of the above factor was an independent risk factor for long‐term mortality; in contrast, high cys C level was an independent predictor of long‐term mortality.

**Table 4:** Multivariate predictors of mortality

Variable	Adjusted OR	95% CI	*P* value
Predictors of all‐cause mortality			
Age	1.07	1.04‐1.11	<0.001
HR on admission	1.02	1.00‐1.04	0.043
Cys C	1.51	1.30‐1.75	<0.001
Predictors of cardiac mortality			
Age	1.05	1.00‐1.09	0.019
HR on admission	1.03	1.00‐1.05	0.036
Cys C	1.36	1.20‐1.53	<0.001

Abbreviations: CI, confidence interval; OR, odds ratio.

## 4 DISCUSSION

This study showed that patients with a higher cys C levels at admission had a higher long‐term mortality; at the same time, they also had a more advanced Killip, lower eGFR, a higher serum creatinine and a higher prevalence of hypertension. After adjusting for these potential confounders, high cys C was an independent predictor of long‐term mortality in STEMI patients receiving late PCI.

STEMI patients undergoing PCI tend to suffer from myocardial ischemia/reperfusion injury, which is a result of interactions between various factors, such as oxidative stress, intracellular Ca^2+^ overload, rapid restoration of physiological pH at the time of reperfusion, and inflammation.^12^ Because cys C modulates inflammatory response, it may play a role in myocardial ischemia/reperfusion injury. Increasing evidences show that cys C is associated with cardiovascular risk and mortality^13^ in patients with non‐ST elevated acute coronary syndrome^14^ and is the most powerful predictor for major adverse cardiac events in patients with acute coronary syndrome.^15^ Akgul et al have investigated the predictive value of elevated cys C in STEMI patients undergoing primary PCI,^16^ and have found that the elevated cys C level, rather than the creatinine level nor eGFR, was an independent predictor of increased in‐hospital and 1‐month cardiac mortality in patients with STEMI undergoing primary PCI.^8^ Tang et al have found that elevated cys C levels at admission were independently associated with impaired myocardial perfusion, poor cardiac functional recovery, and development of congestive heart failure in these patients.^10^ A meta‐analysis showed that cys C was strongly and independently associated with subsequent risk of myocardial infarction.^13^ However, the prognostic role of cys C specifically in STEMI patients undergoing late PCI remains unclear. Our data showed that elevated cys C level at admission was independently associated with long‐term mortality in STEMI patients undergoing late PCI. Identifying patients at higher risk as early as possible is of clinical significance and would allow close observation and positive intervention, and therefore, improve clinical outcomes.

It is not surprising that older patients have much higher levels of cys C in our study. Older patients often have high blood pressure and age‐related diseases such as renal failure.^8^ A previous study has shown that hypertension and renal failure are more common in patients with high cys C.^17^ Our results also showed that the high cys C group had a higher incidence of hypertension, while a lower level of EGF. Produced by nearly all nucleated cells, cys C levels are independent of age, sex, or muscle mass.^18^ Cys C is freely filtered by the glomerular, with no secretion and tubular reabsorption,^19^ and is considered a marker to evaluate renal function. The high cys C level can be used to identify early eGFR abnormalities and is a highly sensitive marker for detecting preclinical renal dysfunction.^20^ There is a close correlation between cardiac diseases and renal diseases.^11^ Previous studies have found that eGFR is inversely associated with cardiovascular events and patients with low eGFR had a significantly higher all‐cause mortality.^21^, ^22^ In our study, eGFR was evaluated by the MDRD equation, which is based on the serum creatinine levels. However, serum creatinine levels are affected by many factors, such as age, gender, muscle mass, diet, and physical activity, and therefore are not sensitive enough to detect mild renal dysfunction.^8^

The exact mechanism of the association between cys C and long‐term mortality has not been clearly elucidated. We propose that two potential countermeasures that may be responsible for the association of high cys C levels with increased long‐term mortality. First, cys C is associated with inflammatory responses.^15^, ^23^, ^24^ Inflammatory responses play an important role in the development of no‐reflow.^25^, ^26^ Therefore, high‐circulation cys C may be associated with a strong cardiac inflammatory response, contributing to the development of no‐reflow and the increased risk of death. Second, high cys C levels are highly sensitive markers for mild renal dysfunction.^6^, ^27^ Mild renal dysfunction is associated with microvascular endothelial dysfunction, which may result in poor myocardial perfusion and clinical outcomes.^28^, ^29^, ^30^

The study is limited in its retrospective and observational nature. The patient age varied broadly and was not balanced between the high and low cys C groups. The results may also confound with LVEF and other unknown or unmeasured factors and are subject to selection bias. In addition, the sample size of the high cys C group is relative small (n = 192), which may raise sampling errors.

## 5 CONCLUSIONS

Patients with high cys C level were older, tended to have hypertension, advanced Killip class, and lower eGFR. High cys C levels are associated with long‐term all‐cause mortality and cardiac mortality after adjustment of these potential cofounders, serving as an independent predictor for risk of cardiovascular events and deaths in STEMI patients treated with late PCI.

## CONFLICT OF INTEREST

The authors declare no potential conflict of interests.

## AUTHOR CONTRIBUTION

Yuewu Chen drafted the manuscript and contributed to data analysis; Yan Fan performed the statistical analysis; Min Men and Guidong Shen collected the data; Aiqun Ma conceived and supervised the study.
